# Negotiating (dis-)engagement in K-12 blended learning

**DOI:** 10.1007/s10639-021-10714-w

**Published:** 2021-08-28

**Authors:** Nina Bergdahl, Melissa Bond

**Affiliations:** 1 Department of Computer and Systems Sciences (DSV), Stockholm University, Stockholm, Sweden; 2 University College London, London, UK

**Keywords:** Engagement, Disengagement, Work pace, Teacher leadership, K-12, Blended learning

## Abstract

It is well-recognised that engagement is critical for learning and school success. Engagement (and disengagement) are, however, also influenced by context. Thus, as digital technologies add complexity to the educational context, they influence classroom leadership, lesson designs and related practices, and thereby engagement. Despite being critical, engagement and disengagement are not well explored concerning these influences, with a lack of research undertaken within socially disadvantaged schools. In this qualitative study, 14 classroom observations were conducted, during five months, in twelve classes in an upper secondary school in Sweden, along with dialogues with teachers (n=12) and students (n=32). The data were analysed using thematic analysis and descriptive statistics. Identified themes include digital context, teacher leadership, engagement and disengagement. A network of relations between the (dis-)engagement compound and themes is presented. The results identified processes in which engagement shifted into disengagement and vice versa; in particular, that the intention of active learning does not automatically translate to active learning for all students, although teachers employed a higher work pace than did their students. Teacher self-efficacy and awareness of how to manage digital technologies in and outside the classroom was found to play a vital role in facilitating engagement. Understanding the (dis-)engagement compound in blended learning environments is key to inform active and visible learning for future research and supportive organisational structures.

## Introduction

Research on the effectiveness of digital technologies shows diverging outcomes. Some conclude that there is potential, but that school digital maturity and teaching practices do not align with the use of digital tools in society at large (Gudmundsdóttir et al., 2014; Håkansson-Lindqvist, 2015; Krumsvik & Skaar, 2020), and that digitalisation does not lead to increased academic achievement (Chen & Jang, 2010; Giesbers et al., 2013; Warschauer et al., 2014). While the literature is replete with research on educational technology and design interventions, approaches have been criticised that overlook axiology, that is; questions of principle and value (e.g. Raes et al., 2020; Tulu et al., 2019), research on engagement in Learning Management Systems (LMS) (Matcha et al., 2019) or evaluating teachers' IT skills (Saubern et al., 2020), with calls for informative examples on how to (better) intervene in the learning environment(s) and use existing digital technologies effectively *in situ*. As education has shifted towards becoming more digital, particularly after the COVID-19 outbreak (see Bond, 2020a; Bond et al., 2021), it is important to understand how teachers and the digital technologies used for learning influence engagement and disengagement. While engagement is often described as the visible and measurable outcome of motivation (Boekaerts, 2016; Fredricks & McColskey, 2012), many teachers report that student disengagement is the biggest challenge they face in their classrooms (Fredricks, 2016). Where engagement is strongly correlated with proactive behaviours for learning, general school success and retention (Bergdahl et al., 2019; Finn & Zimmer, 2012; Wylies & Hodgen, 2012), disengagement is related to disruptive behaviours, negative attitude, withdrawal, absenteeism and school dropout (Alexander et al., 2001; Greener, 2018; Griffiths et al., 2012). However, engagement and disengagement are malleable (Fredricks et al., 2004; Fredricks et al., 2019), and thus teachers, learning environments and digital technologies (and the various uses of them) may influence engagement (Bond & Bedenlier, 2019).

Due to the strong relationship between engagement and disengagement to either school success or school failure (Finn & Zimmer, 2012; Ma et al., 2015), insights into how teachers are utilising digital technologies within blended learning environments and how these, in turn, influence engagement and disengagement are critical to schools (Hietajärvi et al., 2015). It remains important to realise that behind school success or failure lies an individual's success or tragedy, which further accentuates the need for a deeper understanding of how engagement and disengagement manifest, are altered or redeemed, alongside teacher considerations or agile didactic decisions in Blended Learning (BL) (Lawson & Lawson, 2020). A growing body of research has found that today's students are becoming increasingly disengaged in school; displaying increased levels of boredom (Salmela-Aro et al., 2016b; Yazzie-Mintz, 2007), taking the opportunity to escape the classroom via digital devices when feeling bored (Bergdahl et al., 2019), along with general passivity, zoning out, and even occurrences of sleeping (Canaleta et al., 2014; Fredricks et al., 2016; Wang et al., 2017), all of which may increase during a pandemic, but then without a teacher present to support the individual student. Recent reports have called for research that can inform the transformation of Emergency Remote Teaching (ERT) into high-quality distance learning (Darling-Hammond et al., 2020) and concluded that there is a lack of research "... with a specific focus on how the use of collaborative learning with the support of digital tools affects socio-economically disadvantaged students" (Swedish School Research Institute, 2020:10). In addition, a systematic review on global responses to the pandemic in secondary education also concluded that disadvantaged students have received very little focus (Bond et al., 2021). This study, therefore, explores the (dis-)engagement compound in BL in a socially disadvantaged school to meet this gap, and contributes to the field as it brings together aspects of teacher leadership, digital technologies and management of engagement and disengagement in a real classroom setting, to explore meaningful facets.​​ More specifically, this paper adds an exploration and further refinement of the (dis-)engagement compound, which has been called for (Chiu, 2021; Ryan & Deci, 2020), where the digital context, work pace, learning design, and teacher self-efficacy are explored in relation to student (dis-)engagement.

Informed by the above, this study seeks to answer the following research questions:

- 1) How do the uses of digital technologies influence how students (dis-)engage in a disadvantaged upper secondary school?
- 2) How does classroom leadership influence (dis-)engagement in a disadvantaged upper secondary school?

## Background

### The blended learning context and the (dis-)engagement compound

BL combines online and physical elements, such as instruction, material, resources, and learning activities (Bonk & Graham, 2012). For the purpose of this article, digital technologies refer to the devices (e.g., laptops, mobile phones), digital resources with learning content, or to support learning activities (e.g., applications for online meetings, *here*: Google Meet), digital infrastructure, (e.g., that include the Internet and overarching learning management systems; here*:* Google work suite for Education), but also other hardware (e.g., cameras, chargers, headphones, projectors). The BL context is thus infused with varying kinds of digital technologies and resources. When entering a classroom (physical or digital), the teacher needs to establish an agreement – a teacher-student contract – which serves to remind and consolidate the structure, expectations, agreements and positions between the teacher and the students. The teachers communicate norms and expectations, explicitly and implicitly. Even if teachers would not establish a teacher-student contract, they cannot separate themselves from the school context and [Blended] learning environment (Bond & Bedenlier, 2019). Kuh (2010) refers to teachers’ negotiation and facilitation of needs as an engagement compound that establishes the roles, structure, expectations, agreements and positions between the teacher and the student. However, while the communication can be coloured by personality, structural and contextual factors, it may also be influenced by the BL context, the digital technology and teacher IT-literacy (Bond, 2020b). While non-pedagogical digital tools and resources may be explored separately from pedagogical digital tools and resources (see for example Rolf et al., 2019), both may influence the (dis-)engagement compound (Bergdahl et al., 2018a, b). The school, the teaching profession, the role of students, and even digital technologies, are not value-free; and individuals and digital technologies are heavily intertwined, which is why teaching cannot be separated from the context in which it happens (Barab & Squire, 2004; Bond & Bedenlier, 2019).

### Teacher leadership

Teacher leadership entails quite a few perspectives. For the purpose of this article, we explore the blended learning context, teacher self-efficacy, and management of student (dis-)engagement in learning.

#### The blended learning context and work pace

Importantly, digital technologies have the potential to disrupt learning, and the maturity of BL can be viewed as moving from ‘enabling and enhancing’ to transforming learning (Bonk & Graham, 2012). Leadership qualities commonly refer to an individual's traits and characteristics, even though the context may trigger and shape leadership qualities (Fors Brandebo, 2020). In BL and online learning, teacher leadership demands the ability to lead with digital and physical tools and resources in both physical and digital learning environments. Digital technologies challenge the spatiotemporal aspects such as pace, place and time in relation to teaching and learning (Johnson et al., 2016), and studies have proposed that teachers' workload could decrease as a result of ‘working smarter not harder’ (e.g. Kaden, 2020; Kim & Asbury, 2020). At the same time, leadership research has proposed that passive destructive leadership or laissez-faire type of leadership can be triggered by contextual factors such as lack of time, pressure and stress, which then impact teachers ability to exert the leadership they otherwise would (Fors Brandebo, 2020).

#### Teacher self-efficacy and the fostering of engagement

A critical perspective relating to engagement and disengagement is self-efficacy. Established as a socio-cognitive theory, Bandura (1977) emphasised that perceived self-efficacy links one's own ability to manage situations. The self-efficacy theory is *the* motivation theory used to study teachers since it was first applied in 1977 (Fives & Buehl, 2016). Teachers' views on their own ability to influence situations thus govern if they ‘can’ and ‘want' to get involved. Regarding the disengagement compound, the negotiation of engagement and disengagement is strongly related to teachers' self-efficacy, as it places the teachers' perceived ability to influence students in focus. A teacher's self-efficacy affects their leadership in the classroom (Tschannen-Moran & Hoy, 2001) and determines if the solution will be implemented (Fives & Buehl, 2016). Teachers’ self-efficacy refers to their beliefs and to what extent they can influence or affect student learning (Valckx et al., 2020). In addition, Hatlevik (2017) concluded that teachers’ self-efficacy is related to their digital competence and uses of digital tools and resources in and for teaching and learning.

Thus, what teachers do in the classroom is related to their efficacy, but their actions also directly affect student engagement and school outcomes (e.g., Ertesvåg, 2019). Perera et al explored teachers' personality profiles (i.e., ordinary, rigid, well-adjusted and excitable) and how those related to self-efficacy, work engagement, and job satisfaction. They found that job satisfaction was the lowest among excitable teachers, ﻿while well-adjusted teachers was found to report significantly higher self-efficacy in relation to classroom management than teachers in the ordinary and rigid subgroups (Perera et al., 2018). As seen above, research have pointed the importance of exploring contextual influences in relation to leadership. Researchers have addressed a similar need when fostering engagement (Engle & Conant, 2002; Shi et al., 2021). Engle and Conant, highlighted that students need support, relevant resources and authority to engage productively, and that shared norms are needed to be able to hold students accountable for their learning engagement and that such guiding principles can inform teaching practices. Understandably, they did not view these in a BL setting. Some twenty years later, Shi et al, proposed that the blended learning setting in particular that needs to be taken into account when trying to engage students (Shi et al., 2021).

### Engagement and disengagement in blended learning

Research has indicated that context affects engagement both sequentially and reciprocally (Wang & Hofkens, 2019) and emphasised that how digital technologies are used, along with the considerations aimed at promoting engagement and redeeming or circumventing disengagement, is critical for learning (Bergdahl & Nouri, 2020). Together with how and when digital technologies are used, a digital context is shaped, which subsequently influences (dis-)engagement (Bergdahl et al., 2020a; Henrie et al., 2015; Ma et al., 2018). Building on previous engagement and disengagement research (Bergdahl et al., 2019, b; Wang et al., 2017) engagement and disengagement in BL can be understood as a multi-dimensional construct, consisting of four dimensions: a behavioural, a cognitive, an emotional and a social dimension, with engagement encompassing pro-learning behaviours, emotions, focus and interaction, and disengagement encompassing negative emotions, maladaptive behaviours and responses.

### Student self-beliefs

It has been suggested that contemporary theories of learning generally include a section about student beliefs about their competence (Cook & Artino, 2016). Schmid and Petko (2019) explored students' beliefs about their capability of using digital technologies and the perceived usefulness of the same. They found that these aspects are often overlooked, even when digital technologies have a significant impact on the learning context. Schmid and Petko suggest that personalized learning using digital technologies has positive effects on students IT-realted beliefs in learning. However, they also found that “freedom of choice” of learning activities with digital technologies had no significant effect on student beliefs, which may indicate the need for instruction, guidance and leadership. While ﻿one aspect of self-belief relates to their competence, another part relates to a student’s sense of belonging, and thoughts about their relationship with teachers and peers. Functional relationships are critical for students to sense that they are a part of a learning community (Bond, 2020a; Ruzek et al., 2016), and identify as a learner together with other learners (Voelkl, 2012). Research has also pointed out that students' self-beliefs also influence *how* they experience factors related to their learning. A recent study (conducted during the pandemic) (Pelikan et al., 2021) showed that students who had a high perception of their competence to a much greater extent than students who perceived that they have low competence, nuanced their answers and pointed to several influencing factors: results, learning process and context, while students with lower self-confidence briefly stated that nothing was good. Similarly to Schmid and Petko (2019), and Pelikan et al. (2021), Bergdahl et al. (2018a) shadowed students across their school week, and concluded that student engagement varied, but the patterns seemed to be more related to how the teacher orchestrated the digital technologies than student interest in specific subjects. In fact, only one student compensated for poor orchestration with a devoted interest in a subject (Bergdahl et al. 2018b).

Students coming together from several cultures may carry with them varying self-beliefs that influence their learning (Chavous et al., 2003; Fryer & Bovee, 2016). Research exploring the digital divide has focused on digital inequality and concluded that different groups (often multi-cultural and socially disadvantaged) might have limited access to digital tools, may have limited IT-literacy, and also indicated that, even when access and literacy exist, some groups do not benefit from the time they invest online (van Deursen & Helsper, 2015), which during the pandemic has also hindered, for example, immigrant groups from benefiting from the shift to online services in society at large (Ramsetty & Adams, 2020). In schools, studies have found that teachers may reduce the use of digital technologies and resources for immigrant students (Gómez-Fernández & Mediavilla, 2019), which may be due to teacher consideration of student wellbeing, as second language students may have difficulty interpreting social cues, or experience their culture as devalued (Bingham & Okagaki, 2012). Moreover, if left unsupported, students' negative self-beliefs may cause their disengagement to spiral, particularly in online learning (Fryer et al., 2014).

## Methodology

In order to explore the (dis-)engagement concept in depth, a qualitative case study was conducted across five months (September 2020 through January 2021). Case studies allow researchers to explore a phenomenon from multiple angles within their “natural setting” (Willis, 2008, p. 212), which enables data triangulation and validation (Yin, 2014).

### Research context

This case study was conducted in an upper secondary school, in a socially disadvantaged area, in one of the larger cities in Sweden. This is an example of purposeful sampling, which is an appropriate method for selecting sites for deep investigation in qualitative research (Creswell, 2012; Patton, 1990). The upper secondary school welcomes students who have poor primary school results, and while some students study to gain eligibility to enrol with a national program, others are enrolled in a more practically oriented apprenticeship program. The school welcomes students all year round, which means that students, who have just arrived in Sweden, could enrol at any time. In this school, the work is structured around teams that work around their dedicated student groups. Each team of teachers includes a dedicated student health team: a school nurse, a counsellor and a special needs teacher. All teachers and students have their own laptops, use GSuite for Education, and have classrooms fitted with projectors.

### Participants

Following approval and informed consent from the school principal, all teachers at the school and their classes were invited to the study, with 12 teachers agreeing to participate, across the following subjects: Swedish as a second language (SSL), English, Mathematics, Chemistry, Geography, Social Sciences and Music. 32 students (year 10-12) also agreed to participate and provided written, informed consent (see Appendix). Information about the study was always provided to the students verbally in easy-to-understand Swedish, with translations most often made with the assistance of the teacher, teaching assistant or peers.

### Ethical considerations and researcher bias

The school principal and all participants provided written informed consent to participate. All respondents were informed of their right to withdraw from the study at any time without questions asked, and that data would be treated in line with current legislation and analysed and reported anonymously. While, the department had prior established connections with the school the observing researcher had not. The researcher remained an impartial observer during all observations, and did not interfere in any of the lessons. During the five months of observations, the school dedicated a room to the researcher, which further enabled the researcher to spend additional time at the school and with the teachers and students. This familiarity may have helped participants to feel more comfortable in the classroom whilst being observed.

### Data collection

Multiple data sources were collected across the five months (September 2020 through January 2021). Classroom observations (n=14) were undertaken and documented using field notes and photos. Then the teachers assisted in suggesting students that could be observed, close to where the first author would sit to enable observation and dialogue (typically at the back of the classroom) (see Appendix B).

#### Identifying work pace

During the first classroom observation, an emerging indication was that teachers worked to influence student work pace (redeem disengagement). This spurred the interest in observing, and making subjective (yet inductively informed) notes on student and teacher work pace respectively. A schema was developed (see Table 1, Appendix). The schema uses the letters A-E to reflect distinct characteristics of work pace. The work pace schema was added to the classroom observation schema and subsequently used to identify student and teacher work pace in relation to uses of digital technologies during class. Even though there is no equal distance between A - E, the categories were arranged so that A reflects higher engagement and E lower. With the purpose to compare teacher and student work pace, in terms of high and low, the observed pace was re-calculated into numeric values (where E = 1 and A = 5).

### Data analysis

Data were analysed using thematic analysis and descriptive statistics, using actions and processes as units of analysis (Braun & Clarke, 2012). After conducting classroom observations, field notes and photos were coded with memos. Thematic analysis focuses on meaning across a data-set. Data were coded using post-it notes, and codes were subsequently arranged into themes, reflecting instances of actions and processes (ibid.). Codes were discussed between the researchers, explored for emergent, unexpected angles, and re-checked against the data. This reflected a combination of two styles of thematic analysis: 1) descriptive (in which data can be used in illustrative ways) and 2) interpretative (Braun & Clarke, 2012). Thematic analysis was used to identify how the themes could be visualised in a network display (thematic map) (Braun & Clarke, 2006). By arranging and rearranging the themes in thematic maps, the visualising can reveal patterns, support conclusions of the analysis, and provide insights into the relationship between the themes (ibid.). To ensure ‘authentic triangulation’, data collected and analysed were verified by the participating teachers (Yin, 2014).

## Results

**Fig. 1:**
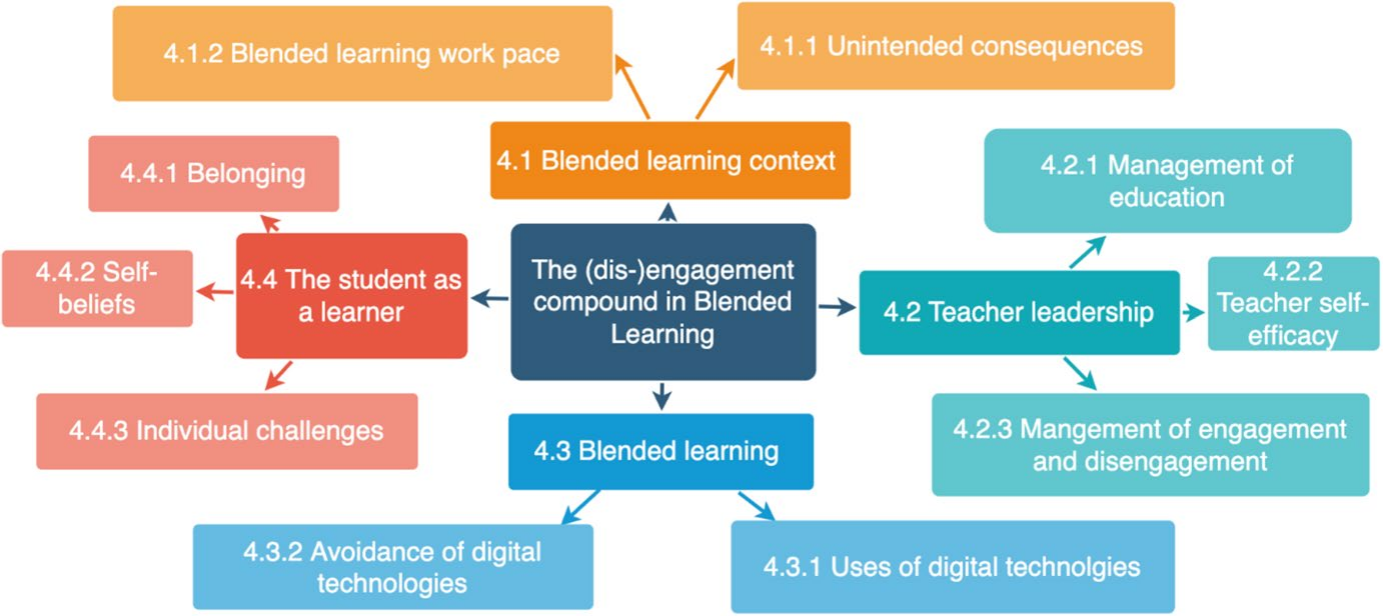
Network of themes identified to influence the (dis-)engagement compound

Figure 1 displays a network of themes related to the (dis-)engagement compound. In exploring the (dis-)engagement compound in BL, four main themes were identified that represent perspectives of influence: 1) The blended learning context, 2) Teacher leadership, 3) Blended Learning activity, and 4) The student as a learner (see Fig. 1).

### The blended learning context

Under the category digital context, two themes were identified: I. Blended learning work pace and II. Unintended consequences.

#### Blended learning work pace

While students could choose which digital technologies to use to work toward their learning objectives, they were also observed to become passive due to using those technologies.

Some students did not have a password and did not have access to the digital learning material - and they continued to be passive throughout the lesson. (Observation 12)

On the other hand, teachers' work pace was high, trying to assist students and encourage them to complete their work.

The teacher was energetic and worked hard to try and meet student needs, while the students were passive. (Observation 2)

The teacher draws on the text for topics and tries to start a dialogue. Students are passive, waiting. The teacher encourages the students further by relating the content to the students' world. (Observation 4)

**Fig. 2:**
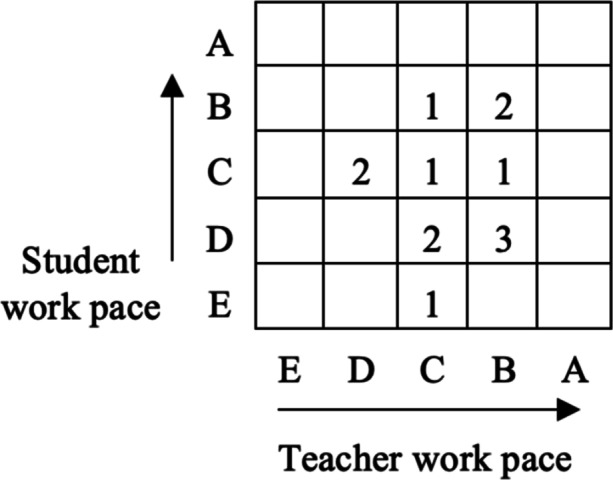
Teacher and student work pace

An emerging indication was that the student pace was observed to be related to and influenced by the teachers’ teaching practices and lesson design. Because of this emerging indication, student and teacher work pace were observed throughout the lessons (see Fig. 2; Table 1, Appendix). All observations included exploring student and teacher work pace in relation to uses of digital technologies during class. In Fig. 2, where category E reflects the lowest work pace and category A the highest. Figure 2 reflects the occurrences of lessons in a certain combination of (student and teacher) work pace. The teachers had in common that their work pace was characterised by categories B, C and D, while the students displayed a larger variety of categories (E, D, C and B), with almost half of the lessons falling into categories reflecting lower pace (E and D) (see Table 1, Appendix for a description of categories).

Figure 2 reveals that some teachers work hard to sustain a high level of activity (category B), but half of the teachers in category B do not succeed in reaching the same level of activity for their students. The teacher pace matched categories C (5) or B (6), with a slight inclination for the higher pace. For students, the most common work pace was identified as categories D (5) or C (4), with the lower work pace being slightly more common. In three observed lessons, teacher and student work pace were the same (fitting categories C and B). Figure 2 also shows that three teachers employed a slower work pace than their students: Teacher: C; Students: B, or Teacher: D and Students C. Neither extreme (E, fully disengaged/asleep or A, stressed to a level of burnout) was observed. The mean of teachers' observed work pace was 3. 30, which was higher than the students’ mean of 2.69. Category A was not observed for teachers or students.

Most often, teachers had a higher working pace than did their students. An emerging indication was that the student pace was observed to be related to and influenced by the teachers’ teaching practices and lesson design. While category E does not include teacher-student interaction focused on learning, designing learning as described in category E may be deliberate for a specific learning goal. However, if the teacher always designs for category E, that decision can reflect values of the surrounding culture and attitudes and may also be a reaction to high work pressure, frustration, and a sense of giving up.

#### Unintended consequences

However, the effect of using educational technology in the classroom meant that students occasionally had to move positions so that they could charge their devices as needed when their batteries ran out, a design consideration that is now essential in modern classrooms. Another effect of using technology was the need for teachers to be cognisant of flexible lesson design, adapting their lesson plans when the Internet, in particular, was not working. When faced with this obstacle, one Music teacher asked the students to turn their computers off and sing instead.

Teacher: "OK - you can turn off your computer now - and we'll sing". (Being online, this would have caused a lesson breakdown.) "Teacher: "Oh, the Internet is up again ... [The teacher can end the lesson by showing streamed media; a snippet of a band who performed the verse that the students had practised]. (Observation 7)

On several occasions, it was observed that the digital technologies the school provided could lead to unintended consequences, such as shared information unintended for students or only being able to listen or use the mobile phone for learning, only if they had access to one.

The teacher logs in to [a digital learning resource] and shows the teacher view... when he does this, all the students can see everything on the teacher's screen as the teacher searches for the information needed for the lesson. (Observation 12)

Teacher: Now we are going to India. This is going to be funny. If you have headphones, you can listen individually [the students use their laptops to log in to an online resource]." (Observation 12)

Other unintended consequences could mean teachesrs had to support students in overcoming challenges, for which there was not always time after class, leading to instruction on how to use IT competing with subject content during lessons.

### Teacher leadership

Three sub-themes of teacher leadership were identified: I. Management of education, II. Teacher self-efficacy and III. Managing engagement and disengagement.

#### Management of education

In some classes, the characteristics of digital technology use related to managing education, such as distribution of learning materials and resources, including directing students to other resources for use outside of class, e.g. students needing to download an app to practice the bass guitar at home. When learning was online, attendance was an automatic feature in another application (e.g., Google Classroom), where timestamps reflected student logins (e.g. lesson 12).

#### Teacher self-efficacy

Teacher self-efficacy includes the motivation to act but may also be influenced by external factors, such as information, organisational support and school culture.

The teacher says he does not know why only nine students are present ... he says that he is very frustrated. There used to be classroom rules posted on the walls in all classrooms. He points to a poster saying, among other things, that mobile phones should be switched off. He sighs and comments that it is not a priority. It is hard not to be heard when you point out that we need to make efforts, such as locking the doors if the students arrive late. Instead, the teacher reports being met with a negative jargon that applies "a certain type of students". (Observation 7)

Teacher self-efficacy is only possible when students are present. On the other hand, views of having ‘a certain type of student’ may reflect a school culture of collective efficacy, impacting individual teachers.

#### Managing engagement and disengagement

Efficacy and knowledge may influence teachers to increase engagement or manage disengagement. The observations, however, included instances when engagement shifted into disengagement and vice versa. In three observed lessons (1, 4, 12), engagement was observed to shift into disengagement. In the observed classes, such instances revealed that instruction to actively work triggered engagement (students got ready to work), but when the teacher continued to talk, instead of allowing work as promised, the students returned to their mobile phone games. In the two other classes, there was little consideration on active learning for all, especially when digital technologies allowed for simultaneous activity, and when the teacher took over from digital technologies, s/he would engage with one or a few students. The majority of students would then be passive.

In five of the observed lessons (1, 5, 7, 9, 10), there were instances when student disengagement shifted into engagement. A low threshold invited students to engage in some lessons (e.g., 10), even after missing classes. Often, little effort was observed that challenged students' cognitive level. Instead, there was a clear focus on raising the low achievers above the pass threshold. However, if lessons are always un-demanding to be inclusive, this risks lowering or omitting the cognitive challenge that spurs progression for the present students.

Nonetheless, the way teachers respond to disengaged students signals teacher expectations and what will be accepted. In one of the lessons (1), it was observed that the teacher ignored the disengaged student and refrained from communicating any fostering norms. This reflects a delicate balance on how and when it may be suitable to approach disengagement, but it may also remain unmanaged. However, there was one teacher that succeeded with students that had failed in other classes.

Teacher: (became teary-eyed) he does not function well in the other classes. And when [another student’s name] first came to me, she was very aggressive. But I talked to her, met her and talked about what she wanted in life and that I empowered. I supported and encouraged her. And now, now she's not like that. She functions very well [in class].” (Observation 9)

When asked about managing disengagement, the teacher forwarded that “it is not about offering treatment - it is about being human, engaging in dialogue and showing you care, that may help turn schooling around fully for students, then the challenges pale in comparison". In other instances, when the teacher-initiated interaction, students were observed to display a range of responses; some students took the initiative to learn, others would rest or even yawn. There was no instruction on what to do when the teacher was interacting with other students.

### Blended learning

Two sub-themes were identified: I. Uses of digital technologies, II. Avoidance of digital technologies.

#### Uses of digital technologies

It was observed that digital and physical resources were often combined and that students were accustomed to bringing both digital and physical tools with them to class; some had pen, paper and books, and others also had laptops and mobile phones.

After the film, the students work on the [digital learning resource] material... They can see the exercise on the whiteboard and their screens. (Observation 12)

A text is projected onto the whiteboard. The students can access the text through Google Classroom. The teacher reads: "Ebba has an exciting book with her and a chocolate bar to munch on. She sinks into the comfortable blue seat on the train. Lisa's mother will meet Ebba. But no Ebba gets off. "Why doesn't Ebba get off the tram?". The teachers remind the students of the built-in audio support: "You can listen to the text again if you have headphones." When the question is raised, students use translation apps, including image search in Google, to visualise what a tram is. (Observation 4,

**Photo 1:**
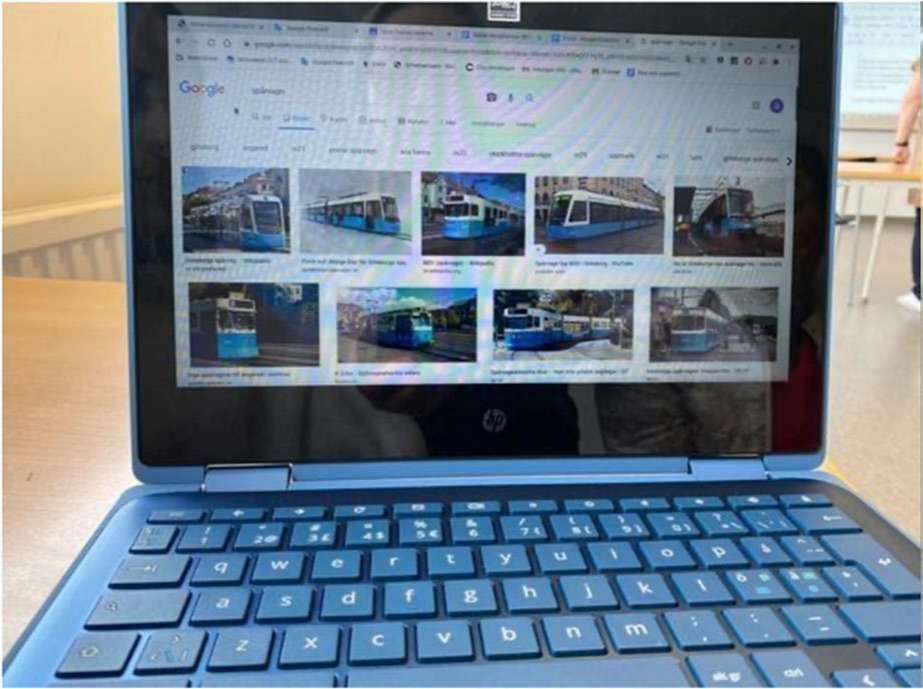
Student using Google image search

**Photo 2:**
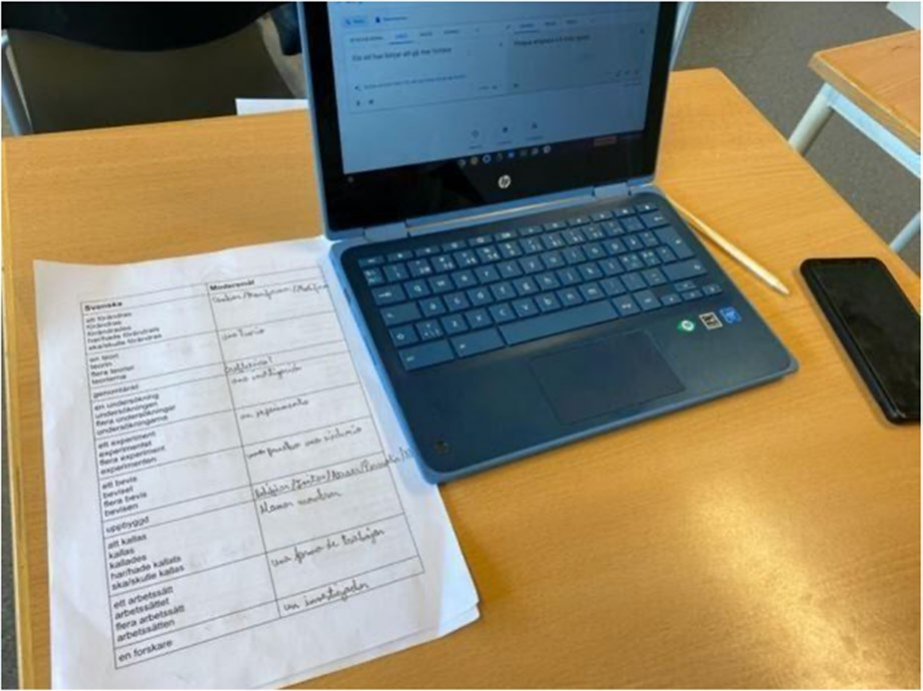
Student using a translation application

Photo 1, 2)

Digital tools seem to be intertwined with the social and culture, that it is expected to function and serve as a foundation from which to manage learning (such as an LMS), distribute tasks (Google Classroom), using media (i.e. access media via individual laptops, or to project imagery or play audio or streaming media to a screen at the front). Digital media has become a standard in classrooms of today.

#### Avoidance of digital technologies

There were also situations when digital technologies (and the properties thereof) were not used. Reasons included that writing by hand was needed or preferred, that digital applications were no longer supported by the developer, and a gap between digital uses during class and school structures enabling student access to digital technologies and digital literacy training.

I have worked with [application name] for eight years … Now, the company no longer releases updates, and I can no longer get an overview of the students’ progression…But I still use the functions I can. (Observation 9)

Teacher: " Today we are not using the computers. It is because it takes a long time for the students to log in. Even if we use [name of application] almost every lesson, it takes too much time from the lesson". (Observation 11)

The digital context appears to frame the conditions under which the digital technologies can be used to both manage education and support learning. There were several instances when digital technologies were the cause of problems: such as teachers having only one device (laptop) or outdated learning resources, which may indirectly affect student engagement negatively.

### The student as a learner

Three sub-themes were identified: I. Belonging, II. Self-beliefs, and III. Individual challenges.

#### Belonging

When a student signals they dare to be passive during an active learning activity, they challenge the teacher and norms. The lesson derives from students making their own choices to engage in learning, with the student having to take his/her initiative to learn. When a student chooses not to make that choice, it can be interpreted as a rebellious protest, a signal that something is not right within him/her. Moreover, it may create an unsettled atmosphere, especially if the student holds a status position in the class.

[Sound from phone]. The teacher does not react. Two students who had arrived late both used their mobile phones. One hides under the desk and spends the lesson time scrolling instead. This behaviour signals shared norms between the latecomers. However, in the class, other norms existed. Another student then takes the opportunity to signal belonging to another set of norms "I have already written everything". This student raises his hand every time the teacher asks a question. (Observation 6)

The disengagement is infectious, spreading to nearby peers. Moreover, if other students have unrest, struggle to concentrate or self-regulate, they may become distracted or take the opportunity to disengage actively. When several outbursts of disengagement happen simultaneously in class, the teachers' stress levels were observed to increase. The teacher could not oversee all behaviour and hence did not set boundaries directed to specific individuals, as they would act out behind the teacher’s back.

#### Self-belief and withdrawal

One teacher described that the school caters for socially disadvantaged students; that some are even accustomed to physical abuse from their teachers. When arriving in Sweden, they are often unsure of the rules and norms in the Swedish classroom. As such, one critical aspect for these students is to re-evaluate their self-beliefs in the new context, where diverse cultures and norms co-exist. Many students were observed to display silence and withdrawal. While self-beliefs may refer to one's ability, it may also reflect insecurities. When the students withdrew into passivity, it appeared as if the passivity had different levels, as if one layer was a temporary idle mode, waiting for the teacher to activate them. The other level, observed when students were left alone for a longer time, was interpreted as if students retracted into a state of isolation and loss of agency, even in class.

When the teacher is with the students, works to engage them, and helps them get started, the students begin to work, but without the teacher's constant prompts, awareness, and leadership, they tend to return to the passive state. (Observation 7)

When the teacher turned to manage disengaged students, the other students in the class received no teaching or instruction. The teachers were observed to balance continuing their teaching or managing student disengagement at the expense of educating the class.

#### Individual challenges

There were instances when teachers tried to engage students and then encountered other challenges, such as student knowledge gaps, or that latecomers could remain invisible in online classes as there was less disruption, which in turn might not trigger a teacher reaction. A third observed challenge was that different students might respond differently to the same situation.

Students were also observed to respond differently to learning activities. In the same situation, one student proactively showed that he was participating in a situation that enabled passive presence. In a similar situation, another student had his eyes open, looked ahead, and observed to work but did not engage in classroom interaction. (Observation 12)

In a classroom, students take on different roles. There might not be room for every individual student to be proactive, who takes the initiative to talk. On the other hand, in classes with no student interaction, little dialogue or momentum, a verbal exchange can challenge the cognitive level.

## Discussion

In answering the first research question: ”How are the uses of digital technologies influencing how students (dis-)engage in a disadvantaged upper secondary school?”, we found that in BL, there were both uses and deliberate avoidance of digital technologies. In the observed BL classrooms, digital technologies were used to enhance learning, often combined with traditional practices. For example, classroom observations revealed that teachers frequently use their laptops to project content onto the whiteboard (see Table 1, Appendix), which resembles the blackboard, but with digital equipment. While projecting content saves time and helps avoid problems that may arise when teachers have to turn their back against students, it remains a traditional approach (Gudmundsdóttir et al., 2014). Teachers would then alternate between engaging in dialogues with students and directing questions to students, referring to the content on display.

Expanding on previous research (Engle & Conant, 2002; Perera et al., 2018), we found that a teacher is physically managing the classroom (to foster engagement) by initiating and shifting interactions (e.g., question/answer, dialogue), tone of voice, signals, prompts, and by providing or withholding information. Our results also show the impact of digital technologies on the learning context e.g., when teachers initiated uses or avoided uses of digital technologies, experienced technology breakdowns or that students lacked necessary digital skills, equipment, or login details. Such contextual occurrences directly affected students' actual possibilities to engage proactively in BL activities and demanded the teacher to shift the order of learning activities promptly. Several teachers also displayed an accumulated understanding of students’ vocabulary, anticipating new and potentially problematic words, and were attentive for cues, particularly related to students not understanding the language. With meticulous perfection, teachers identified students' level of knowledge, or expanded on students' insights, using gestures, tone of voice, visualisations and peer translations to communicate their message. However, in a digital learning context, relying on non-verbal communication, gestures and enthusiasm, is far from the design-thinking needed in a BL context. Merely offering a traditional class online may then cause some unexpected disturbances. For example, whilst turn-taking was an accepted method a couple of decades ago, it may be interpreted as un-demanding, slow and boring for the students of today, who may look to be simultaneously active using digital resources. Indeed, when relying on traditional ways of teaching, seven out of ten students were passive, and four out of these seven did not accept sitting passively but either initiated private conversations or turned to their mobile phones. That is, the very interaction that used to be effective, or at least accepted in the classroom some decades ago, was promoting passivity and reduced interaction (e.g. Luckin, 2008).

In regards to question two: “How does classroom leadership influence (dis-)engagement in a disadvantaged upper secondary school?”, classroom leadership was found to influence (dis-)engagement directly. Results indicate that teacher leadership in BL entails both self-efficacy and the knowledge of how to design lessons that positively influence engagement and work pace and manage disengagement. As expected, ICT tools and related activities were used in almost all lessons. Still, the tools were almost always treated as something that should be handed over to the students to choose and utilise, without moderation or prior consideration by the teacher. In line with Valckx et al. (2020), we agree that teachers' beliefs of what they can do to influence student learning relate to their self-efficacy. However, apart from leadership efficacy (Tschannen-Moran & Hoy, 2001), we argue that digital self-efficacy is needed. There were differences in how ICT tools and related activities were used amongst the teachers, and there was no indication that access to technology, or students lacking digital literacy, would hinder implementing BL. On the contrary, students used many technologies as an integrated part when switching between their educational and privacy spheres (Giesbers et al., 2013; Rashid & Asghar, 2016; Zheng & Warschauer, 2015).

ICT tools were standard when it came to teachers managing the class and the content. However, they were rarely used in a deliberate way to shape learning or engagement. Warschauer et al. (2014) argued that how teachers integrate ICT tools and pedagogical thinking, aiming to promote engagement and learning, is critical, with teachers in this school seemingly making the same considerations throughout their lesson planning. On the one hand, it is understandable that ICT knowledge differs. On the other, developing an overall school strategy could lead more teachers to develop their use of ICT for pedagogical purposes (Boekaerts, 2016; Furrer & Skinner, 2003). Developers of educational technologies should incorporate axioms, considering what teachers need and how those can be met, and considering that poor designs may trigger unwanted behaviours, considering whether learning will be increased, alongside the actual benefits of the software will be. The conditions to exert leadership is influenced by critical contextual factors (Fors Brandebo, 2020). Thus, the digital context, work pace, designs for learning and teacher self-efficacy are viewed as critical aspects of the (dis-)engagement compound. Bergdahl et al. (2018b) concluded that engagement can be designed for if teachers are supported in becoming more aware of their potential to influence student (dis-)engagement. Extending these findings, the results found that teachers' planning seemed to be teacher-centred and that active learning is not active learning for all students. When all students were expected to engage in learning and supported by lesson design actively, students were not observed to disengage.

A lack of digital devices or outdated applications was also observed to impede teacher excellence. However, structural access needs to be combined with digital competence and awareness of implications that occur when shifting between the teacher and the digital tools and resources as agents for learning. When using digital technologies, a teacher could serve an unlimited number of students, as digital technologies are used to mediate one-to-many communication. In this case, though, when the teacher withdrew the digital technologies and turned to just one or a few students, the majority of students were left passive, and few students knew what to do. This resulted in most students being left passive for up to three times longer than the duration of observed technology breakdowns. Moreover, when comparing student and teacher work pace, teachers often worked at a more intense pace than their students (Kaden, 2020; Kim & Asbury, 2020). This implies that digital competence is more than subject didactics, or how to teach one's subject using digital resources and IT skills. We suggest that IT competencies should include aware considerations of consequences relating to digital technologies and digital leadership. Indeed, passive or disruptive presence negatively affected the learning climate for students and the working climate for teachers. The teachers were struggling with managing and redeeming disengagement, and often, individual or even collective denial was observed. Coming to class and being actively disengaged may be a students' way of repeating negative self-beliefs; that no one cares. In the online classes, there was considerably less disengagement and managing of maladaptive behaviours. However, considering the uneven digital competencies and teachers struggling to offer support (see Fryer et al., 2014; Fryer & Bovee, 2016), not only wanting to give support but ensuring school structures adapt to the needs of support appears critical. In BL learning classes, it also became important how the teacher worked to establish a positive learning climate or react to disengagement signals. However, working with negative self-beliefs remains a challenge (Fryer et al., 2014; Fryer & Bovee, 2016).

## Conclusion

Building on the analysis and previous work, the results indicate that negotiation of engagement in the BL setting is a legitimate problem as the (dis-)engagement compound is affected by both individual traits and context. Thus, a complex network of factors seem to influence learners (dis-)engagement.

- First the school context, which includes teacher workload with factors like time, pressure and stress that may negatively impact the conditions to realise positive leadership.
- Second, digital technologies were found to influence leadership conditions. Thus, we propose that teachers’ classroom leadership should include digital self-efficacy, and teachers’ IT competencies should include digital leadership.
- Third, results indicate that teachers' work pace was related to awareness of the impact of digital technologies, alongside a teacher's digital awareness of how to orchestrate digital technologies and resources to reduce work pace.
- Moreover, results show that teachers manage (dis-)engagement quite differently: reveal shifts both to engagement from disengagement, and vice versa. Therefore, the (dis-)engagement compound can be understood as interactions within the BL context, the conditions for teaching and learning, and leadership execution, the learning activities and students' beliefs, sense of belonging and individual challenges.

Thus, the negotiation of student (dis-)engagement, in a BL context, relates to teacher self-efficacy; namely, their beliefs about their ability to influence students' (dis-)engagement, teacher work pace, whether affected by digital competence or other stressful factors, influencing conditions to exert leadership, knowledge on how to design for engagement, and manage disengagement.

### Limitations and future work

Generalisations from this study are limited. First, the sample size and number of schools are insufficient to generalise the conclusions. Second, the study was conducted in one school in a socially disadvantaged area. More students, schools and diversity of the same should be selected for increased generalisability. Moreover, despite observing several classrooms and lesson, any such observation does at best provide a snippet of that teacher’s practice, and those students’ engagement. Future research could explore negotiations in other BL settings or use a longitudinal design. The findings do however contribute to the research field, in terms of proposing instances and occurrences when digital technologies hinder and promote engagement, describe how engagement may shift into disengagement and vice versa, and point to the need to include managing in digital context as a skill for teachers. Future research should explore teachers’ digital leadership and digital self-efficacy, learning designs, teacher considerations in relation to online and blended learning.

## Appendix

### Teacher and student work pace

Table 1 describes characteristics of categories A-E reflecting teaching practices in BL classrooms and student practices from a perspective of pace.

**Table 1:** Teacher and student pace (without digital technologies)

Cat.	Student work pace	Teacher work pace	The role of digital technologies
A	Stressed. The lesson is characterised by constant high pace and demands where the goals are perceived as unreachable, for example when the teacher provides multiple tasks, unclear instructions, no support or resources and too little time.	The lesson is characterised by lack of digital leadership, and high levels of perceived stress for teachers, where they feel overwhelmed and/or exhausted.	Characterised by worry or anxiety related to using digital technologies, in combination with limited digital competence in relation to error detection, troubleshooting and problem solving, (which may be enhanced by limited school support, and/or a conviction that digital technologies have no place in education).
B	Higher pace that requires concentration. The lesson is characterised by intentionally higher pace, which enables diverse student pace and a need for student autonomy where several activities or variations of activities are ongoing.	The lesson is characterised by agile teaching practices. The teacher works to activate the students in each learning activity.	Characterised by competing elements of teacher attention which may cause stress, such as new, unfamiliar or non-functional software or digital devices, increased workload due to creating new content, assessments and exams, flipped classrooms, feedback. The work pace may increase as teachers try to exert control, when the teacher experiences disengaged students, and/or a lack of structural support. Stressed teachers can talk faster to "survive the lesson or class", dreading to be there.
C	Active learning which sometimes is cognitively challenging. The lesson is characterised by students either engaging in hands-on practice or working to master a theoretical content. The majority of students are kept at this pace.	The lesson is characterised by flexible teaching practices. The teacher works to activate the students in each learning activity.	Characterised by re-using video-annotations, flipped classrooms, visual feedback, and designs for gamification. Re-using online self-assessment and quizzes with randomised answers and questions and auto correction. Reusing designs that previously worked to enable a variety of peer interactions using cloud services and online forums.
D	Slow and repeated instances of enabling passivity. Characterised by empty slots between learning activities/learning sequences, or one activity that did not last for the duration of the lesson.	The lesson is characterised by few or fragmented activities that do not overlap smoothly, or too few learning activities, leaving students who finish tasks early in a passive space without instructions.	E.g. the focus on the lesson design derives from a self-centred approach: "what do I do as a teacher", rather than how are the students actively involved in learning, while I am focusing on “xyz”. Digital technologies are not used to provide exercises, enable interactions or challenge students cognitively.
E	Slow and unpretentious. Characterised by low design effort. The teacher does not support a work pace directed toward learning, and allows for a minimum of learning sequences. The lesson signals that if truant or absent, you have not missed anything.	The lesson is characterised by non-agile presence. This lesson can include passive "laissez-faire style of teaching”, but it can also be, aware design, where the teacher deliberately designs the lesson to decrease their own work pace, and increase the students' work pace	E.g. redirecting students to learn from a source separate from teacher interaction such as a movie, animation, streamed media, working on a project or use digital devices to enable dialog, interaction or training.

## Declarations

### Data availability

Anonymised results are available from the authors by request.

### Code availability

n/a
